# Educational achievement and youth homicide mortality: a City-wide, neighborhood-based analysis

**DOI:** 10.1186/s40621-020-00246-1

**Published:** 2020-06-08

**Authors:** Michael J. C. Bray, Mary E. Boulos, Galen Shi, Kevin MacKrell, Paul S. Nestadt

**Affiliations:** 1 Department of Psychiatry, Johns Hopkins University School of Medicine, Baltimore, MD USA; 2 Michael G. DeGroote School of Medicine, MacMaster University, Hamilton, Ontario Canada

**Keywords:** Youth homicide, Education, Gun violence, Homicide, Violence, Injury prevention, Community health, Neighborhood safety, Health inequality, School policy

## Abstract

**Background and objective:**

Educational achievement, particularly among youth, may mitigate risk of exposure to violence and negative related health outcomes such as crime and gang activity. Few studies to date have examined relationships between education and youth homicide. The authors hypothesized association between educational achievement in grades 3 and 8 and youth homicide mortality.

**Methods:**

Neighborhood-based, city-wide analysis was conducted of cross-sectional data regarding *N* = 55 neighborhoods in Baltimore, MD, extracted from Baltimore 2017 Neighborhood Health Profiles.

**Results:**

Higher educational achievement (operationalized by reading proficiency) in third, but not eighth, grade was associated with reduced neighborhood youth homicide mortality rates in hierarchical linear regression, controlling for demographic and socioeconomic factors (*ß* = − 0.5082, *p* = 0.03), such that each 1.97% increase in proportion of students reading at an acceptable level was associated with one fewer neighborhood youth homicide per 100,000. Neighborhoods within the highest tertile of youth homicide mortality differed from those in the lowest tertile with fewer males (45% vs. 48%, *p* = 0.002), greater unemployment (17% vs. 8%, *p* < 0.001), familial poverty (35% vs. 16%, *p* < 0.001), and residents identifying as black or African-American (88% vs. 25%, *p* < 0.001). Causal mediation analysis demonstrated mediation effects of familial poverty and eighth grade educational achievement through third grade educational achievement (ACME = 0.151, *p* = 0.04; ACME = − 0.300, *p* = 0.03, respectively) with no significant direct effects.

**Conclusions:**

Higher educational achievement (operationalized by reading proficiency) predicts reduced homicide mortality among Baltimore youth and appears to mediate effects of familial poverty on homicide mortality as well. This converges with literature highlighting the importance of education as a determinant of social capital and violence. Future policy-based interventions should target inequalities in educational achievement to mitigate homicide risk among youth in communities facing disparities in violent crime.

## Introduction

Youth homicide is among the most common preventable causes of death in the United States. With firearm homicide representing the third greatest cause of injury death among American youth aged 10–24 (Dahlberg et al., 2015; David-Ferdon et al., 2013), better understandings of mitigating and exacerbating factors are needed to develop targeted interventions. Firearm homicide rates among youth in the United States are substantially higher than any other industrialized nation, exceeding the next leading 25 countries combined (The World Bank, n.d.). At particular risk are youth who are African-American, aged 20–24, or male (David-Ferdon et al., 2013). Furthermore, evidence suggests that individual and neighborhood level poverty further increases risk of exposure to violence and engagement in violent behaviour among youth (McAra & McVie, 2016). Convergently, economic inequality is predictive of neighborhood homicide rates, accounting for variance beyond that attributable to race or other sociodemographic factors (Krueger et al., 2004).

Among youth, death by homicide is a complex biopsychosocial phenomenon likely mediated through myriad associated factors. In addition to race and economic status, familial structure is a notable determinant of homicide risk, with both maternal and paternal absence predicting higher community rates of homicide (Schwartz, 2006). Similarly, longitudinal investigation of state-level trends has found increasing prevalence of single-parent families is correlated with increased rates of homicide mortality across the United States (Amato & Patterson, 2017). At the neighborhood level, communities with a greater proportion of single-parent families bear higher rates of homicide mortality, controlling for race and income-to-needs ratio (Krueger et al., 2004). Importantly, single-parent families bear greater economic need and insecurity, yet reap similar welfare support compared to two-parent families (Garfinkel & Zilanawala, 2015). This economic inequity compounds psychosocial challenges faced by single-parent families, producing high collinearity between familial structure and other risk factors, including poverty (Jones-DeWeever et al., 2003).

Family structure and neighborhood characteristics may be difficult to change, however, enhancing educational achievement represents a potentially modifiable target, which may serve to mitigate homicide risk among youth. Multiple investigations have reported strong associations between both educational engagement and achievement and subsequent violence exposure (Borofsky et al., 2013; Jones-Webb & Wall, 2008; Steffensmeier et al., 2010). Literature suggests high educational achievement facilitates reductions in adult homicide rates both through reduced vulnerability to violence (Hazekamp et al., 2018) and decreased risk of violent behavior (Herrenkohl et al., 2012). However, while many consider educational achievement to be protective against violence and homicide mortality (Totten, 2009), previous studies have demonstrated mixed results. In certain communities, though potentially confounded, increasing prevalence of residents achieving a high-school education or greater has been accompanied by rising rates of youth homicide (Hazekamp et al., 2018). In high-school students, Pardini and colleagues reported that, while high academic achievement was associated with unadjusted reductions in violent behaviour amongst youth aged 15–18, this was not significant after controlling for psychiatric and sociodemographic factors (Pardini et al., 2012).

Though much remains to be elucidated, education remains an important consideration concerning youth homicide, warranting further study. This is particularly true regarding African-American communities, which, despite equivalent educational aspiration, bear disproportionate burdens of poor educational achievement in addition to youth homicide risk (Lee et al., 2011). Notably, racialized achievement gaps in education begin as early as kindergarten and persist throughout one’s educational career, exerting potentially life-long impact (Curran & Kellogg, 2016). It is hopeful that interventions targeting racialized educational inequalities might also show efficacy in reducing racialized inequalities regarding youth homicide.

While youth homicide represents a far-reaching, national concern, certain cities bear particularly elevated risk for death among children and adolescents. With 55.77 homicides per 100,000 inhabitants, Baltimore, Maryland has the second highest homicide rate of any city in the United States (Crime in the united states, 2018). Notably, homicide rates increased substantially nation-wide between 2014 and 2015, with Baltimore experiencing a 58.5% increase in homicide mortality. This increase exceeds any increase in the city’s history since 1985 (Rosenfeld, 2016). The high burden of homicide mortality borne by youth in Baltimore’s heterogenous neighborhoods presents an important opportunity to evaluate determinants of risk, and mitigating factors, with broader implications for similar communities nationally. Therefore, the present investigation sought to examine educational achievement as a mediator of homicide mortality among youth residing in Baltimore neighborhoods. The authors hypothesized greater educational achievement would be associated with reduced rates of youth homicide mortality.

## Methods

### Data collection

Data was obtained from the Baltimore City Health Department’s 2017 Neighborhood Health Profile Reports (Baltimore City Health Department, 2017). These reports provide data regarding health, social environment, built environment, and demography for *N* = 55 discrete neighborhoods comprising Baltimore, MD. Neighborhoods are identified using *Community Statistical Areas*: discrete geographical regions defined by the Baltimore City Planning Department with boundaries reflective of residents’, institutions’, and city planners’ perceptions of community boundaries. All neighborhoods were included for analysis. This study was acknowledged by the Johns Hopkins University School of Medicine Institutional Review Board (IRB00206312).

Data was extracted regarding the following variables: gender composition, racial composition, familial poverty, unemployment rate, proportion of single-parent households, adult educational achievement, school absenteeism (elementary), school absenteeism (middle school), educational achievement (third grade), educational achievement (eighth grade), and youth homicide rate. Racial composition, familial poverty, unemployment rate, proportion of single-parent households, and adult educational achievement data were extracted from the American Community Survey’s 2011–2015 five-year estimates (U.S. Census Bureau, 2015). School absenteeism and educational achievement were extracted from the Baltimore Neighborhood Indicators Alliance via the Baltimore City Public School System for the 2013–2014 academic year.

Variables were operationalized as follows:

#### Racial composition

Proportion of neighborhood residents self-identifying as black or African American.

#### Familial poverty

Proportion of neighborhood families with children under 18 years of age reporting annual household income below the poverty level.

#### Unemployment rate

Proportion of unemployed residents aged 16 and older in the civilian labour force.

#### Proportion of single-parent households

Proportion of neighborhood children aged 18 or younger living in single-parent households.

#### Adult educational achievement

Proportion of residents aged 25 years or older who have obtained a high school degree or less.

#### School absenteeism

Proportion of students in elementary (first-fifth grades) or middle school (sixth-eighth grades) reported to miss 20 or more days of school in the previous school year.

#### Educational achievement

Proportion of third or eighth grade students assessed to have reading levels of “Proficient” or “Advanced” by the standardized Maryland School Assessment exam. Possible rankings include “Advanced,” “Proficient,” and “Basic.” Third and eighth grades were chosen due to application of standardized testing during these academic periods. Notably, in Maryland, students are not given the option of opting out of these assessments as they are in some other states.

#### Youth homicide mortality rate

Rate of death due to homicide occurring per 100,000 youths under the age of 25 (based on victim residence) between 2010 and 2014, comprising a total of 425 homicide events. Youth mortality data were reported by the Maryland Department of Health and Mental Hygiene’s Vital Statistic Administration.

### Statistical analysis

Analysis was performed using R statistical analysis software version 3.6.1. Descriptive statistics were calculated, and a correlation matrix was constructed using Pearson correlation coefficients. Primary hypothesis testing was performed using mixed-effects, least-squares regression modeling. Comparative analysis of neighborhoods comprising upper and lower tertiles regarding the primary outcome variable (youth homicide rate) was performed using Welch’s T-tests. Detection of problematic multicollinearity among individual predictor variables was performed using variance inflation factor (VIF) calculation, corrected VIF (CVIF) calculation (Curto & Pinto, 2011), and Leamer’s method (Leamer, 1973). Threshold for VIF and CVIF was set a priori at ≥10 (Salmerón et al., 2018). Overall multicollinearity detection was performed by calculation of the determinant (D) of the correlation matrix (threshold set a priori at D ≤ 0.01) and sum of inverse eigenvalues (threshold set a priori at 25, 5 times the number of predictors) (Chatterjee & Haldi, 2006). For each possible pairing of predictors used in multivariate regression, causal mediation analysis was performed using 1000 simulations of non-parametric bootstrapping (confidence intervals calculated via percentile method) and youth homicide as the primary outcome. Each neighborhood was included for analysis as one data point and therefore all were equally weighted concerning calculations of means and other statistics, regardless of differing population size. Therefore, presented means represent mean neighborhood rate and do not reflect Baltimore’s global means.

## Results

Mean neighborhood demographic composition was 47% male, 62% black/African-American, 31% white, and 2% Asian with 5% of Hispanic ethnicity (Table 1). Wide ranges were observed regarding proportions of students with high educational achievement in both grade 3 and grade 8, with mean values of 59 and 59% respectively. Average neighborhood youth homicide rate was found to be 28.18/100,000 individuals younger than 25 annually. Other neighborhood characteristics are presented (Table 1).

**Table 1:** Descriptive statistics of demographic, socioeconomic, and educational characteristics of *N* = 55 neighborhoods in Baltimore, Maryland. Welch’s T-Tests comparing demographic and socioeconomic characteristics of neighborhoods in the upper and lower tertiles with regards to youth homicide rate are presented

Variable	Mean (SD)	Range	Highest Tertile (n = 18) Mean	Lowest Tertile (*n* = 18) Mean	*T*	*df*	*p*-Value
Gender Composition (% Male)	46.9 (2.6)	(41.3–54.1)	45.31	48.23	− 3.479	26.192	**0.002**
Racial Composition (% Black)	61.8 (32.8)	(2.5–98.6)	88.23	25.09	11.198	23.848	**< 0.001**
Racial Composition (% White)	31.4 (29.3)	(0.5–90.1)	8.02	63.05	−9.864	21.61	**< 0.001**
Racial Composition (% Asian)	2.5 (3.0)	(0.0–13.9)	0.89	5.34	−4.913	20.077	**< 0.001**
Ethnic Composition (% Hispanic)	4.9 (5.8)	(0.0–31.9)	1.92	8.36	−3.342	18.403	**< 0.001**
Familial Poverty Rate	27.9 (15.9)	(2.6–63.3)	35.22	15.86	4.697	33.512	**< 0.001**
% Unemployment	13.9 (6.5)	(2.3–26.4)	17.45	7.76	5.913	33.999	**< 0.001**
% of Children in Single-Parent Homes	62.6 (22.6)	(11.2–94.3)	77.16	39.9	6.773	26.975	**< 0.001**
% of Children with High Educational Achievement in Grade 3	59.2 (14.8)	(34.5–97.1)	51.4	70.36	−4.713	24.876	**< 0.001**
% of Children with High Educational Achievement in Grade 8	58.7 (13.0)	(40.7–94.3)	51.7	70.73	−5.353	26.874	**< 0.001**
% of Adults with a High School Degree or Less	47.4 (17.7)	(6.9–72.0)	57.33	30.95	5.533	29.001	**< 0.001**
Elementary School Absenteeism	13.7 (5.3)	(0.4–24.8)	16.26	9.51	4.385	32.623	**< 0.001**
Middle School Absenteeism	15.4 (5.6)	(3.7–29.9)	17.31	13.49	2.281	32.974	**0.03**
Youth Homicide Rate	28.2 (21.9)	(0–107)	53.03	6.47	11.146	20.637	**< 0.001**

### Comparison of neighborhoods with high vs. low youth homicide rates

Educational and socioeconomic characteristics of neighborhoods in the highest and lowest tertiles regarding youth homicide were compared (Table 1). Neighborhoods with high youth homicide rates (upper tertile) had higher proportions of black/African-American residents (88% vs. 25%), with lower proportions identifying as white (8% vs. 63%), Asian (1% vs. 5%) or Hispanic (2% vs. 8%) and lower proportions of male residents (45% vs 48%), compared to neighborhoods with low youth homicide rates (lowest tertile). Neighborhoods with higher youth homicide rates also differed with higher familial poverty rates (35% vs. 16%), unemployment (17% vs. 8%), percentage of children in single-parent homes (77% vs. 40%), proportion of adults with a high school degree or less (57% vs. 31%) and elementary and middle school absenteeism (16% vs. 10 and 17% vs. 13% respectively), as well as lower rates of educational achievement in grades three and eight (51% vs. 70 and 52% vs. 71% respectively). All differences reported above were significant at the *p* < 0.05 level.

### High early educational achievement is associated with lower youth homicide rates

Table 2 displays zero-order, Pearson correlations between variables of interest. Strong correlations between predictor variables highlight the possibility of high collinearity. Only middle school absenteeism and neighborhood racial composition were not significantly associated. Relevant to the primary hypothesis, neighborhood rates of educational achievement in grades three and eight were highly correlated with youth homicide rates (*p* < 0.001).

**Table 2:** Zero-order, Pearson correlation matrix of demographic, socioeconomic, and educational characteristics of Baltimore City neighborhoods as well as rates of youth homicide

	1	2	3	4	5	6	7	8	9	10
1. Race	–	–	–	–	–	–	–	–	–	–
2. Familial Poverty Rate	**0.6042**^Ɨ^	–	–	–	–	–	–	–	–	–
3. Unemployment Rate	**0.7332**^Ɨ^	**0.7553**^Ɨ^	–	–	–	–	–	–	–	–
4. Single-Parent Rate	**0.8309**^Ɨ^	**0.7928**^Ɨ^	**0.7884**^Ɨ^	–	–	–	–	–	–	–
5. % of Children with High Educational Achievement, Grade 3	**−0.5801**^Ɨ^	**−0.724**^Ɨ^	**−0.6429**^Ɨ^	**−0.7228**^Ɨ^	–	–	–	–	–	–
6. % of Children with High Educational Achievement, Grade 8	**−0.7226**^Ɨ^	**− 0.7484**^Ɨ^	**− 0.7058**^Ɨ^	**−0.8686**^Ɨ^	**0.7747**^Ɨ^	–	–	–	–	–
7. Adult Educational Achievement	**0.6669**^Ɨ^	**0.7345**^Ɨ^	**0.8422**^Ɨ^	**0.7671**^Ɨ^	**−0.7142**^Ɨ^	**−0.7445**^Ɨ^	–	–	–	–
8. School Absenteeism (Elementary)	0.4874***	**0.6341**^Ɨ^	**0.5977**^Ɨ^	**0.6408**^Ɨ^	**−0.5991**^Ɨ^	**−0.6887**^Ɨ^	**0.7008**^Ɨ^	–	–	–
9. School Absenteeism (Middle School)	0.1171	0.3248*	0.3255*	0.3468**	−0.3631**	−0.4007**	0.3052*	**0.6448**^Ɨ^	–	–
10. Youth Homicide Rate	**0.7114**^Ɨ^	0.5000***	**0.6080**^Ɨ^	**0.6704**^Ɨ^	**−0.5953**^Ɨ^	**−0.5855**^Ɨ^	**0.5993**^Ɨ^	0.4936***	0.2799*	–

^Ɨ^***p*** < 0.00001, ****p < 0.0001, ***p < 0.001, **p < 0.01, *p **< 0.05**

Table 3 displays unadjusted, linear regression modeling. Educational achievement in both grades 3 and 8 displayed significant, negative associations with neighborhood rates of youth homicide (*p* < 0.001). This was further evaluated using hierarchical, mixed-effects linear regression modeling (Table 4). In block one, race was entered into the model. Racial composition subsumed substantial variation with a model *R*^*2*^ of 0.506, such that a higher proportion of black residents was associated with higher neighborhood rates of youth homicide (ß = 0.4745, *p* < 0.001). In block two, familial poverty and single-parent rate were entered into the model. While both showed high zero-order correlation with youth homicide rates (Table 2), neither accounted for significant variation after adjusting for race. In block three, educational achievement in the third and eighth grade were entered into the model. Notably, higher third grade educational achievement was significantly associated with lower youth homicide rates after adjusting for race, familial poverty and proportion of children in single-parent homes (ß = − 0.5082, *p* = 0.03). Eighth grade educational achievement was not significant after adjusting for socioeconomic variables.

**Table 3:** Unadjusted regression model evaluating key variables of interest as predictors of neighborhood youth homicide mortality rates

	Unadjusted Model			
	ß	95% CI	T-Value	*p-*Value
Race	0.4745	(0.3483–0.6007)	7.369	**< 0.001**
Familial Poverty Rate	0.6870	(0.3665–1.0075)	4.203	**< 0.001**
Single-Parent Rate	0.6480	(0.4549–0.8411)	6.578	**< 0.001**
Educational Achievement, 3rd Grade	−0.8818	(−1.2023 - -0.5613)	−5.394	**< 0.001**
Educational Achievement, 8th Grade	−0.9832	(−1.3497 - -0.6167)	−5.258	**< 0.001**

**Table 4:** Hierarchical linear regression model examining effects of educational achievement on youth homicide rates after controlling for demographic, socioeconomic, and familial factors

	Model 1				Model 2				Model 3			
	ß	95% CI	*T*	*p-*Value	ß	95% CI	*T*	*p-*Value	ß	95% CI	*T*	*p-*Value
*Block 1 (R*^*2*^ *= 0.506)*												
Race	0.4745	(0.3483, 0.6007)	7.369	**< 0.001**	0.3311	(0.1017, 0.5605)	2.829	**0.007**	0.3333	(0.1097, 0.5569)	2.920	**0.005**
*Block 2 (R*^*2*^ *= 0.5264)*												
Familial Poverty Rate					−0.0164	(−0.4479, 0.4150)	−0.075	0.94	−0.1712	(− 0.6216, 0.2792)	−0.745	0.46
Single-Parent Rate					0.2585	(−0.1762, 0.6931)	1.165	0.25	0.2397	(−0.2576, 0.7370)	0.945	0.35
*Block 3 (R*^*2*^ *= 0.5679)*												
Educational Achievement, 3rd Grade									−0.5082	(−0.9674, − 0.0490)	−2.169	**0.03**
Educational Achievement, 8th Grade									0.2747	(−0.4172, 0.9666)	0.778	0.44

### Multicollinearity detection

VIF and CVIF were calculated for all predictor variables included for multiple regression analysis. Both VIF and CVIF were < 10 for all variables. Leamer’s method similarly detected no problematic multicollinearity for any predictor variable. Regarding measures of overall collinearity, the sum of inverse eigenvalues was calculated as 22.16 suggesting no problematic collinearity. However, the determinant of the correlation matrix was calculated as 0.0093 exceeding the predetermined threshold of D ≤ 0.01, highlighting possible model multicollinearity.

### Causal mediation analysis

**Fig. 1:**
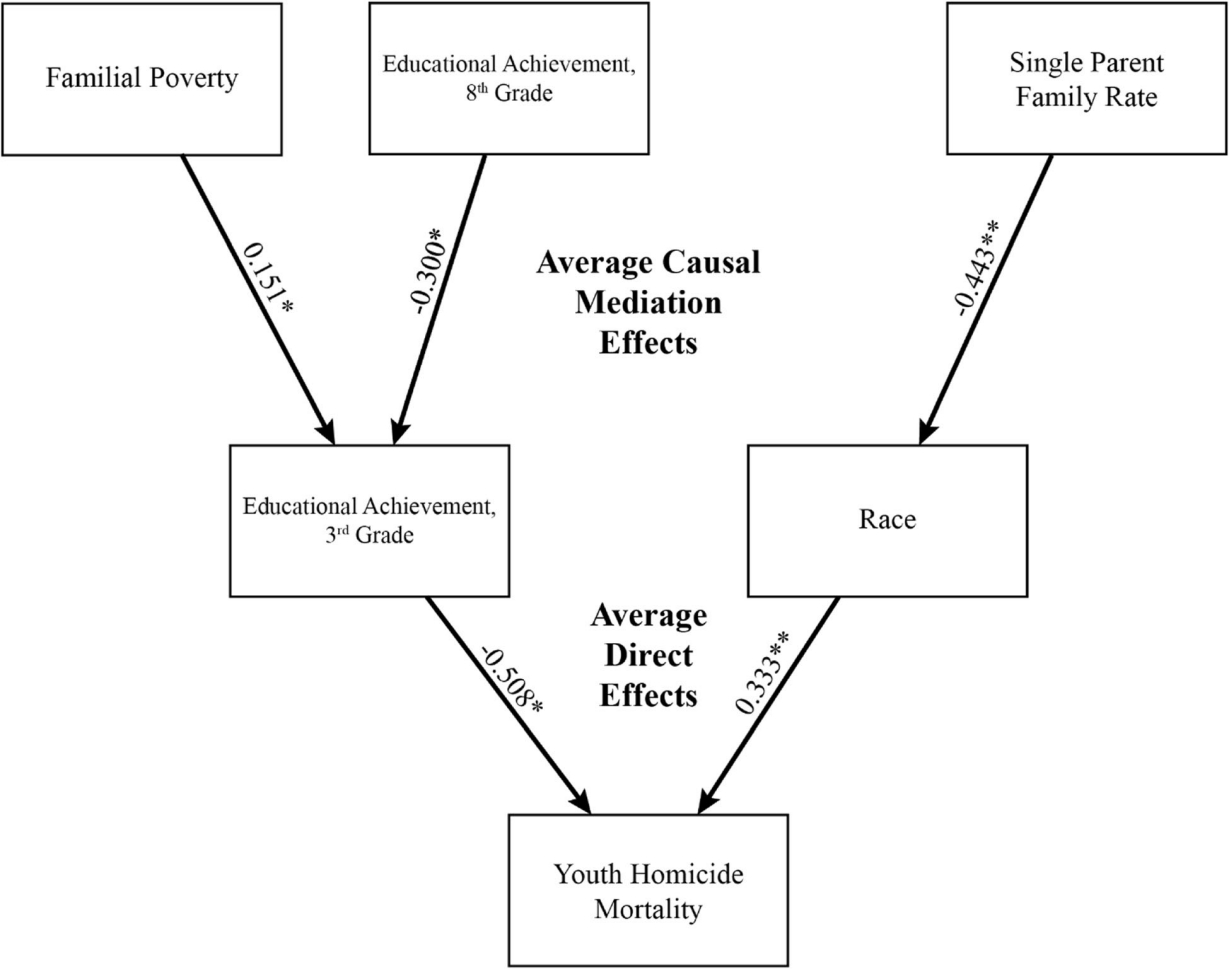
Path diagram of average causal mediation effects and average direct effects of variables of interest on neighborhood youth homicide mortality rates. Effects of familial poverty and educational achievement, 8th grade, are shown to be fully mediated through educational achievement, 3rd grade, while effects of single parent family rate are shown to be fully mediated through race. No direct effects of familial poverty, educational achievement, 8th grade, or single parent family rate were found to be significant by causal mediation analysis. **p < 0.01, *p **< 0.05**

Results of causal mediation analysis are displayed in Table 5. Significant mediation effects were identified for all predictor variables which did not reach the threshold for significance in the adjusted model. Effects of familial poverty rates and eighth grade educational achievement regarding youth homicide mortality were fully mediated through third grade educational achievement (average causal mediation effects [ACME] = 0.151, *p* = 0.04; ACME = − 0.300, *p* = 0.03, respectively) with no significant direct effects, while effects of single-parent rate were fully mediated through race (ACME = − 0.443, *p* = 0.01) with no significant direct effects. Significant direct effects with no significant mediation effects were seen for race (average direct effects [ADE] = 0.333, *p* < 0.01) and educational achievement (Grade 3) (ACME = 0.508, *p* = 0.03). Path diagrams are presented (Fig. 1).

**Table 5:** Summary of causal mediation analyses determined through non-parametric bootstrapping (1000 simulations per analysis). Direct and causal mediation effects of each independent variable through possible mediator variables are described in terms of effect on the primary outcome variable, neighborhood youth homicide mortality rate, controlling for other model variables. ADE: average direct effects; ACME: average causal mediation effects

	*Mediator*	1. Race		2. Familial Poverty Rate		3. Single-Parent Rate		4. Educational Achievement, 3rd Grade		5. Educational Achievement, 8th Grade	
*Independent Variable*		ACME	ADE	ACME	ADE	ACME	ADE	ACME	ADE	ACME	ADE
1. Race		–	–	0.013	0.333**	0.065	0.333**	−0.003	0.333**	−0.003	0.333**
2. Familial Poverty Rate		−0.103	−0.171	–	–	− 0.103	−0.171	0.151*	− 0.171	−0.010	− 0.171
3. Single-Parent Rate		0.443**	0.240	−0.079	0.240	–	–	0.009	0.240	− 0.094	0.240
4. Educational Achievement, 3rd Grade		0.007	−0.508*	0.053	−0.508*	−0.003	− 0.508*	–	–	0.071	− 0.508*
5. Educational Achievement, 8th Grade		−0.039	0.275	0.015	0.275	−0.159	0.275	−0.300*	0.275	–	–

**p < 0.01, *p **< 0.05**

## Discussion

To our knowledge, this is the first study suggesting early educational achievement mitigates neighborhood-level youth homicide mortality. We found higher educational achievement in third grade was associated with lower youth homicide rates, such that each 1.97% increase in proportion of students reading at an acceptable level was associated with one fewer neighborhood youth homicide per 100,000, adjusting for race, familial poverty and proportion of children in single-parent homes. Eighth grade educational achievement was not a significant predictor, adjusting for demographic and socioeconomic variables. However, significant mediation effect was observed for eighth grade educational achievement through third grade educational achievement. This may suggest that, while educational achievement exerts a protective effect regarding youth homicide mortality, academic success in grade three predicts success in grade eight, as early educational achievement is a known determinant of later-life educational achievement (Reynolds et al., 2001). Future work is required to further elucidate these complex relationships.

Tests of problematic multicollinearity were largely negative, suggesting results are unlikely to be substantially impacted by inflated variance due to multicollinearity. However, it should be noted that the calculated determinant of the correlation matrix surpassed the pre-defined threshold of D < 0.01 by a small margin (D = 0.0093). Mediation analysis suggested that effects of single-parent rate were mediated through race, while effects of familial poverty rate and eighth grade educational achievement were mediated through third grade educational achievement. This likely accounts for much of the observed collinearity and should be considered when interpreting null findings in multiple regression models.

The present study suggests early educational achievement represents a modifiable factor which may serve to reduce risk of youth homicide within communities. Previously, Pardini and colleagues examined effects of educational achievement on violence in youth aged 13–18 enrolled in Pittsburgh public schools and found no significant effect after controlling for demographic and socioeconomic variables (Pardini et al., 2012). Similarly, Hazekamp and colleagues examined the impact of high school educational achievement in Chicago on youth homicide rates and found that youth homicide continued to increase locally despite increases in educational achievement (Hazekamp et al., 2018). The authors suggested the contagious nature of violence, spreading through social networks, undermines education’s protective effect during this time, highlighting the importance of reducing violence exposure during this vulnerable period (Hazekamp et al., 2018). Perhaps consistent with these findings, the present investigation did not find significant direct effects of eighth grade educational achievement in adjusted models. Our study extends these findings by demonstrating notable association between earlier educational achievement (third grade) and youth homicide mortality, with significant mediation effects of grade eight exerted through grade three.

This finding converges with a wealth of literature suggesting early education exerts substantial, positive impact on various, later-life outcomes. Meta-analysis of early education interventions in the United States has demonstrated benefits regarding cognition, school progress, social development, and emotional development (Camilli et al., 2010). Separate meta-analysis of international early education interventions demonstrated similar effects in cognitive, behavioral and school progress-focused domains (Barnett, 2011; Nores & Barnett, 2010). Furthermore, educational achievement mitigates future risk of poverty among at-risk youth (Abelev, 2009). Notably, early childhood education interventions are associated with reduced later-life delinquency, including arrests for violent crimes (Reynolds et al., 2011; Reynolds et al., 2001). Collectively, this suggests that early education interventions may offset homicide risk through social developmental, familial, and community factors, with far-reaching benefits breaking cycles of poverty and violence (Noguera, 2011; Wright et al., 2016).

Highlighting mechanistic probability, literature suggests protective effects of early educational achievement are mediated through various developmental factors, including improved social adjustment, motivational advantage, and cognitive advantage (Reynolds et al., 2004). Similarly, higher educational achievement in early childhood is associated with improved behavioral self-regulation and social competence (Liew, 2012). These correlates of early educational success may exert long-lasting effects on both homicide risk and subsequent eighth grade educational achievement.

Effects of familial poverty were found to be fully mediated through third grade educational achievement. Poverty has long been robustly associated with poorer educational outcomes, with children in resource-poor communities bearing reduced access to educational resources; a discrepancy related to lower familial resources and reduced funding of neighborhood schools (Jackson et al., 2016; Payne & Biddle, 1999). While poverty is a known determinant of educational achievement, it is also known to be associated with increased violence exposure (Krueger et al., 2004; McAra & McVie, 2016). The present findings suggest that this detrimental effect of poverty may be mitigated by improving early education outcomes. Although future study is needed, it is possible that interventions targeting early educational achievement may mitigate exacerbating effects of poverty regarding youth homicide mortality. Importantly, relationships between youth homicide and early educational achievement may be bidirectional, with higher rates of neighborhood violence presenting barriers to academic success (Macmillan & Hagan, 2004). Compellingly, violence exposure is associated with poor educational outcomes among youth. Examining homicide rates, Caudillo & Torche found that exposure to neighborhood violence increased risk of academic failure among elementary school students (Caudillo & Torche, 2014). This may be related to secondary behavioural problems in classroom settings, which have been correlated with exposure to community violence (Goeke-Morey et al., 2013). Moreover, this impact may be extended within classrooms, exerting far-reaching effects on other children, as classes containing students from families with reported history of domestic violence have been reported to score lower on tests of reading and math compared to those without, within the same school (Carrell & Hoekstra, 2010). Finally, it is notable that African American students exposed to violence are at particular risk of poor educational outcomes, with a higher risk of dropping out than their Caucasian peers, following violence exposure (Peguero, 2011).

On comparison of upper and lower tertiles, neighborhoods with the highest rates of youth homicide were comprised of a smaller proportion of males as compared to neighborhoods in the lowest tertile (45.31% vs. 48.23%). This is consistent with past work describing the phenomenon of “missing men,” whereby socioeconomically disadvantaged communities, particularly those comprised largely of African American residents, bear increased rates of incarceration and death among males (Lane, 2004). With men bearing disproportionate homicide risk, this impacts gender compositions of communities with high homicide rates, leading to lower proportions of male residents as compared to communities with fewer homicides (Fox & Fridel, 2017; Gilbert & Ray, 2016). Furthermore, risks of homicide and incarceration are highly correlated at the neighborhood level and incarceration may compound effects of elevated homicide risk on gender composition in the present sample (Gilbert & Ray, 2016; Renauer et al., 2006).

Neighborhoods with higher youth homicide rates also differed with elevated unemployment (17% vs. 8%) and elementary and middle school absenteeism (16% vs. 10 and 17% vs. 13% respectively). This is concordant with previous literature demonstrating associations between employment and reduced risk of homicide mortality among youths aged 18–24 (Davila et al., 2010). Additionally, exposure to neighborhood violence has been previously associated with elevated chronic school absenteeism (≥15 days absent in the past year) (Stempel et al., 2017). Neighborhoods in the upper tertile of youth homicide also bore higher rates of familial poverty (35% vs. 16%), percentage of children in single-parent homes (77% vs. 40%), proportion of adults with a high school degree or less (57% vs. 31%), as well as lower rates of educational achievement in grades three and eight (51% vs. 70 and 52% vs. 71% respectively). All comparisons listed were significant at the *p* < 0.05 level. Collectively, these data highlight trends of systemic disadvantage, whereby neighborhoods at the greatest risk of violence bear disproportionate socioeconomic challenges. In the present study, these challenges comprise numerous consequential variables which influence one another, as suggested by causal mediation analysis. Thus, while the demonstrated association between education and reduced rates of homicide is encouraging and may help delineate solutions to community violence, policy-based interventions must consider the myriad contributing factors to homicide risk and this underlying disadvantage.

These findings converge with previous studies suggesting youth homicide is a complex biopsychosocial phenomenon, linked to a long history of disadvantage involving familial structure, socioeconomic status, and educational achievement in marginalized communities (Borofsky et al., 2013; Jones-Webb & Wall, 2008; Krueger et al., 2004; Schwartz, 2006). Importantly, previous policies designed to alleviate poverty have unintentionally compounded challenges faced by single-parent families, increasing economic instability (Garfinkel & Zilanawala, 2015; Varshney et al., 2016). For instance, though it decreased average poverty rates, barriers to accessing the Temporary Assistance to Needy Families block grant reduced income amongst the poorest tenth of single mothers by 18% when it replaced the Aid to Families with Dependent Children program (Trisi & Sherman, 2016). Policy makers should be acutely aware of the manner in which community and familial factors determine an intervention’s long-term impact. The context in which youth homicide occurs is crucial to understanding and correcting the disproportionate burden borne by marginalized populations. Though improving educational outcomes has proven challenging, particularly in urban environments bearing myriad other socioeconomic inequalities, the present study suggests resources directed towards early educational achievement may mitigate risk of violence among youth; perhaps with greater efficacy than less evidence-based approaches such as expanded incarceration infrastructure (Roeder et al., 2015).

### Limitations

This study bears key limitations which should be considered when interpreting the present findings. First, the number of variables accounted for using hierarchical linear regression modeling is constrained by the present sample, in which analysis at a neighborhood level, as compared to an individual level, produced reductions in statistical power. While this provides valuable insight into neighborhood-level factors influencing rates of youth homicide, future work may build on these findings by examining individual-level data also. Important control variables which were not included for analyses may include gun ownership and community prevalence of lead paint, which is common in poorer Baltimore neighborhoods and may lead to increased impulsivity and subsequent risk of exposure to violence (Carpenter & Nevin, 2009). Similarly, community alcohol availability and proportion of vacant houses correlate with homicide rates and might be considered in future investigations (Jones-Webb et al., 2008; Trangenstein et al., 2018). Importantly, community prevalence of psychiatric dysfunction, particularly substance dependence disorders, may increase both victimhood and perpetration regarding violent crime, simultaneously creating barriers to educational achievement (Seeman & Göpfert, 2004; Varshney et al., 2016; Wilcox, 1985). Elucidating roles of mental health in these complex relationships represents a high priority for future research. Furthermore, inference of causality is limited by study design. We were also unable to control for effects of resident movement between neighborhoods and the ways in which this mediates risk. Finally, Baltimore represents an enriched sample regarding homicide, and therefore, while providing valuable context within which to study mediators of youth homicide, conservative interpretation of results should be exercised when generalizing findings to other contexts.

## Conclusion

Youth homicide is a leading cause of death for adolescents in the United States. Enhancing quality of early education for youth in communities facing disparities in youth homicide may alleviate the burden of violence borne by these neighbourhoods, though policy makers should be aware of substantial barriers to modifying educational outcomes within the context of urban inequality. These findings are relevant to public health debates about the importance of robust educational programs, particularly in disadvantaged communities, to prevent youth violence.

## Data Availability

Aggregated neighborhood-level data was extracted from the 2017 Baltimore City Neighborhood Health Profiles (https://health.baltimorecity.gov/neighborhoods/neighborhood-health-profile-reports). The dataset generated from the extraction of key variables of interest from the 2017 Baltimore City Neighborhood Health Profiles is available from the corresponding author on reasonable request.
